# Plasmablasts as Translational Biomarkers in Autoimmune Diseases: From Cellular Dynamics to Clinical Decision-Making

**DOI:** 10.3390/cimb48010077

**Published:** 2026-01-12

**Authors:** Muhammad Soyfoo, Julie Sarrand

**Affiliations:** Department of Rheumatology, Hôpital Erasme, Université Libre de Bruxelles (ULB), 1070 Brussels, Belgium; julie.sarrand@ulb.be

**Keywords:** plasmablasts, biomarkers, autoimmune diseases, B cells, rituximab, systemic lupus erythematosus, Sjögren disease, IgG4-related disease, precision medicine, flow cytometry

## Abstract

B cells are key drivers of immune dysregulation across systemic autoimmune diseases. Among their progeny, plasmablasts occupy a uniquely revealing niche: short-lived, highly proliferative intermediates that mirror real-time B-cell activation. Their appearance in peripheral blood integrates antigenic stimulation, cytokine-driven differentiation, and aberrant germinal-center dynamics, transforming them into sensitive indicators of ongoing immunological activity. This review synthesizes current knowledge on plasmablast biology and highlights disease-specific phenotypes across systemic lupus erythematosus (SLE), primary Sjögren disease (pSjD), IgG4-related disease (IgG4-RD), ANCA-associated vasculitis (AAV), and rheumatoid arthritis (RA). We incorporate molecular insights from single-cell technologies that have uncovered previously unrecognized plasmablast subsets, metabolic states, and interferon-related signatures with prognostic and mechanistic value. Beyond descriptive immunology, plasmablasts are emerging as dynamic biomarkers capable of informing real-time clinical decisions. One of the most robustly supported applications is the prognostic interpretation of plasmablast kinetics following B-cell-depleting therapies, where early reconstitution patterns consistently predict relapse across multiple autoimmune conditions. As clinical immunology shifts from static serological markers toward kinetic, cell-based monitoring, plasmablast quantification offers a path toward precision immune surveillance. Integrating plasmablast dynamics into routine care may ultimately allow clinicians to anticipate disease flares, time therapeutic reinforcements, and transition from reactive management to preventive intervention.

## 1. Introduction

B cells have progressively gained prominence as central orchestrators of immune dysregulation across systemic autoimmune diseases [1,2,3]. Once viewed primarily as antibody factories, B cells are now recognized as key drivers of antigen presentation, cytokine production, and the dynamic propagation of chronic inflammation [4,5]. The success of B-cell-depleting therapies such as rituximab across multiple autoimmune conditions has underscored the pathogenic relevance of the B-cell compartment and stimulated intense interest in understanding the nuances of B-cell biology in disease [6,7].

Within this complex network, plasmablasts occupy a uniquely revealing position. As short-lived, highly proliferative progeny of activated B cells, plasmablasts provide a real-time biological readout of ongoing immune activation [8,9,10]. Their emergence in peripheral blood reflects active differentiation pathways that integrate antigenic stimulation, cytokine signaling—particularly type I interferons and interleukin-21 (IL-21)—and dysregulated germinal center reactions [11,12]. Unlike long-lived plasma cells that reside in protective bone marrow niches and maintain stable antibody titers over decades [13,14], plasmablasts represent the dynamic, transient arm of humoral immunity [15,16]. Importantly, plasmablasts do not exist in isolation but participate in complex bidirectional interactions with other immune cell subsets, including T follicular helper cells, dendritic cells, monocytes, and regulatory T cells, which together shape the magnitude and quality of humoral responses [4,5].

Over the last decade, advances in flow cytometry, mass cytometry (CyTOF), and single-cell multi-omics technologies have redefined plasmablasts as biomarkers of exceptional translational value [17,18]. Across systemic lupus erythematosus (SLE), Sjögren disease (SjD), IgG4-related disease (IgG4-RD), ANCA-associated vasculitis (AAV), and subsets of rheumatoid arthritis (RA), plasmablast expansion correlates with disease activity, flare onset, and therapeutic response [19,20,21]. In particular, the kinetics of plasmablast reconstitution after B-cell-depleting therapy such as rituximab has emerged as one of the most powerful predictors of relapse, often surpassing traditional serological markers in predictive accuracy [22,23,24,25].

The clinical implications of these observations are profound. Whereas autoantibody titers represent a historical record of immune activation—reflecting the cumulative output of long-lived plasma cells—plasmablast levels provide a window into current immunological activity. This kinetic advantage translates into earlier detection of impending flares, more precise timing of retreatment, and the potential for preventive rather than reactive therapeutic interventions. The emergence of therapies specifically targeting plasmablasts and plasma cells, including anti-CD38 monoclonal antibodies and BCMA-directed agents [26,27], further elevates the clinical relevance of understanding plasmablast biology.

This review examines the biology of plasmablasts, their disease-specific phenotypes, and their expanding role as dynamic biomarkers guiding real-time clinical decisions. We discuss how integrating plasmablasts into routine care could shift immune monitoring from static serology to kinetic precision immunology, and we propose practical algorithms for clinical implementation.

## 2. Biological Overview of Plasmablasts

### 2.1. Definition and Phenotypic Markers

Plasmablasts occupy a transitional stage between activated B cells and long-lived plasma cells [1,2]. They are typically defined by expression of CD19+CD27+CD38+ high, loss of surface IgD, and increasing expression of transcription factors IRF4, Blimp-1 (encoded by PRDM1), and XBP1 [3,28]. Their proliferation index is among the highest in the immune system, with a half-life of only 24–72 h in circulation [8]. This brevity distinguishes them fundamentally from long-lived plasma cells, which can persist for decades in bone marrow niches [13,14].

**Figure 1:**
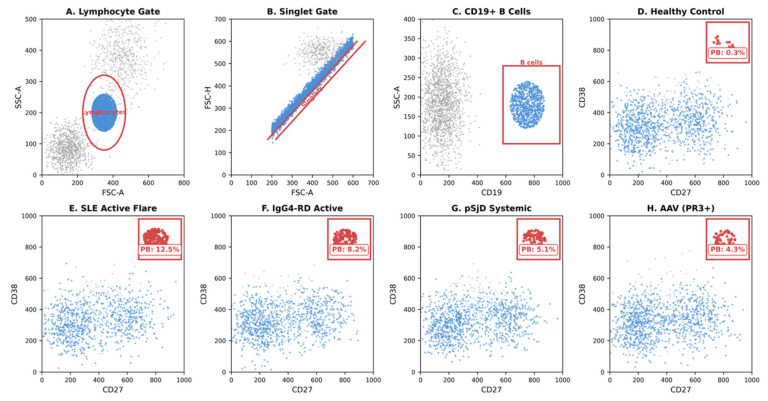
Flow cytometry gating strategy for plasmablast identification in peripheral blood. This figure illustrates the sequential gating approach used to identify and quantify plasmablasts from whole blood samples. (**A**) Initial gating on forward scatter (FSC) versus side scatter (SSC) to identify the lymphocyte population, excluding debris and large cells. Doublet exclusion is performed using FSC-A versus FSC-H to ensure single-cell analysis. (**B**) Within the lymphocyte gate, B cells are identified as CD19-positive cells. (**C**) Plasmablasts are defined within the CD19+ population as cells expressing high levels of both CD27 and CD38 (CD19+CD27+CD38+). The double-positive population in the upper right quadrant represents circulating plasmablasts. (**D**–**H**) Representative flow cytometry plots comparing plasmablast frequencies in healthy controls versus patients with active autoimmune diseases: (**D**) healthy control showing < 1% plasmablasts within B cells; (**E**) active SLE showing markedly expanded plasmablast population (typically 5–20% of B cells during flare); (**F**) IgG4-RD demonstrating elevated plasmablasts; (**G**) pSjD with moderate plasmablast expansion correlating with ESSDAI; (**H**) AAV showing variable plasmablast elevation particularly in PR3-positive disease. The gating strategy follows recommendations from EuroFlow consortium standardized panels. Absolute plasmablast counts can be calculated by multiplying the percentage by the total CD19+ B-cell count [30,31,32].

The surface phenotype of plasmablasts exhibits both conserved and context-dependent features [10,12]. Core markers include high CD38 expression (typically 10-fold higher than activated B cells), bright CD27 positivity, and variable CD19 expression that diminishes as cells mature toward plasma cell identity. CD20 expression is typically low or absent, explaining why plasmablasts are relatively spared during initial rituximab therapy but are affected by CD19-directed agents [29,30]. Additional markers of clinical relevance include CD138 (syndecan-1), which increases with maturation; HLA-DR, which remains high on plasmablasts but decreases on mature plasma cells; and Ki-67, reflecting their proliferative state [31,32,33]. A standardized flow cytometry gating strategy is illustrated in Figure 1.

Unlike long-lived plasma cells residing in bone marrow niches, plasmablasts remain migratory and responsive to immunological cues [15]. They express chemokine receptors including CXCR4 and CXCR3 that direct their trafficking to inflammatory sites and bone marrow. Their abundance fluctuates rapidly in response to antigen exposure, cytokine stimulation, and local inflammation, making them exquisitely sensitive indicators of immune perturbation [16,17,18].

### 2.2. Differentiation Pathways

Plasmablasts arise via two principal pathways with distinct immunological characteristics and clinical implications [5,34,35]:

1/Germinal center (GC) pathway: This canonical pathway is induced by T follicular helper cells (Tfh), IL-21, CD40 ligand (CD40L), and sustained antigen presentation [5,36]. Within germinal centers, B cells undergo iterative cycles of somatic hypermutation and affinity-based selection, yielding high-affinity, class-switched immunoglobulins. Plasmablasts emerging from this pathway typically produce antibodies of superior quality and contribute to protective immunity. The process requires approximately 1–2 weeks and generates both plasmablasts destined for short-term antibody production and precursors of long-lived plasma cells [2,37].

2/Extrafollicular pathway: This rapid differentiation route bypasses germinal center reactions and is driven by Toll-like receptor (TLR) activation, B-cell activating factor (BAFF), a proliferation-inducing ligand (APRIL), and interferon-rich environments [35,38,39]. Plasmablasts can emerge within 3–5 days via this pathway, enabling rapid antibody responses to pathogens. However, the extrafollicular pathway is also prominently active in autoimmune diseases, particularly SLE, SjD, and during viral infections [40,41]. Antibodies produced via this pathway undergo less affinity maturation and may exhibit broader—and potentially autoreactive—specificities.

**Figure 2:**
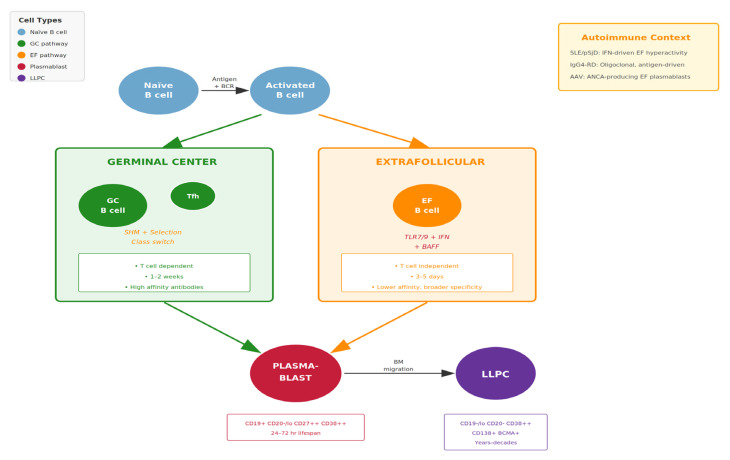
B-cell differentiation pathways in health and autoimmunity highlighting plasmablast generation. This schematic figure illustrates the two principal pathways generating plasmablasts and their relevance to autoimmune disease pathogenesis. In the germinal center (GC) pathway (left), naive B cells activated by antigen and T-cell help enter germinal centers where they undergo somatic hypermutation and affinity maturation over 1–2 weeks. Tfh cells provide critical IL-21 and CD40L signals. This pathway generates high-affinity, class-switched antibodies and contributes to both short-lived plasmablasts and long-lived plasma cell precursors [5,35,36]. In the extrafollicular (EF) pathway (right), B cells differentiate rapidly (3–5 days) without germinal center transit, driven by TLR activation, BAFF/APRIL, and type I interferons. This pathway is hyperactive in autoimmune diseases, particularly SLE and pSjD, due to chronic interferon exposure, TLR7/9 stimulation by nuclear autoantigens, and elevated BAFF levels [34,37,38,39,40,41]. The balance between these pathways varies across autoimmune diseases: SLE and pSjD show prominent EF pathway activation with interferon-high plasmablast signatures [41,42,43,47,48]; IgG4-RD demonstrates oligoclonal expansion suggesting antigen-driven GC responses [49,50]; RA features both pathways with ELS in synovium supporting local plasmablast generation [51,52].

The balance between these pathways has significant implications for autoimmunity. In SLE, the extrafollicular pathway is hyperactive, driven by chronic type I interferon exposure and TLR7/9 stimulation by nucleic acid-containing immune complexes [35,42,43,44]. This results in continuous generation of plasmablasts producing pathogenic autoantibodies. Understanding which pathway predominates in individual patients may guide therapeutic selection, as different agents target distinct aspects of B-cell activation and differentiation [40,45,46] (Figure 2).

### 2.3. Plasmablast Interactions with Other Immune Cell Subsets

**Figure 3:**
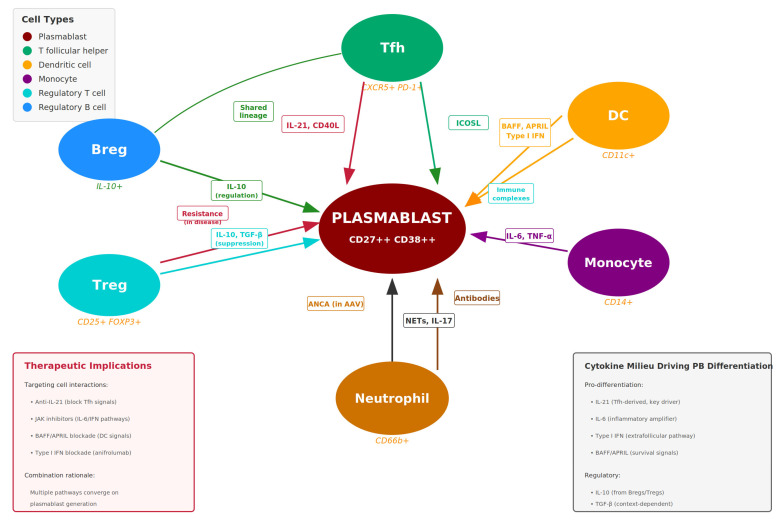
Plasmablast Interactions Within the Immune Network) depicts bidirectional crosstalk between plasmablasts and other immune cells that shapes plasmablast generation, survival, and pathogenic potential in autoimmune diseases. Central plasmablast interacts with: Tfh cells (IL-21, CD40L signals); Dendritic cells (BAFF, APRIL, type I IFN); Monocytes (IL-6, TNF-α); Neutrophils (NETs, IL-17; ANCA axis in AAV); Tregs/Bregs (IL-10, TGF-β regulatory signals). Therapeutic implications box identifies anti-IL-21, JAK inhibitors, BAFF/APRIL blockade, and type I IFN blockade as targets. depicts the bidirectional crosstalk between plasmablasts and other immune cell populations that shapes plasmablast generation, survival, function, and pathogenic potential in autoimmune diseases. Central Plasmablast. The plasmablast (large red circle, center) is positioned at the hub of multiple immune interactions. Plasmablasts are defined by the surface phenotype CD27^++^CD38^++^ and represent short-lived, highly proliferative antibody-secreting cells derived from activated B cells. T Follicular Helper Cells (Tfh; green, top). Tfh cells (CXCR5^+^PD-1^+^) provide critical signals for B-cell differentiation via IL-21 and CD40 ligand (CD40L), which drive plasmablast generation through the germinal center pathway. In return, plasmablasts and their precursors express ICOSL, which sustains Tfh cell function, establishing a positive feedback loop that is hyperactive in diseases such as SLE. Dendritic Cells (DC; orange, upper right). Dendritic cells (CD11c^+^) produce B-cell activating factor (BAFF), a proliferation-inducing ligand (APRIL), and type I interferons (IFN-α/β) that promote plasmablast survival and extrafollicular differentiation. Reciprocally, plasmablast-derived antibodies form immune complexes that are internalized by DCs, further amplifying interferon production—a pathogenic cycle prominent in SLE and primary Sjögren disease. Monocytes (purple, lower right). Monocytes (CD14^+^) contribute to the inflammatory milieu through secretion of IL-6 and TNF-α, cytokines that enhance plasmablast differentiation and survival. Antibodies produced by plasmablasts can activate monocytes via Fc receptor engagement, perpetuating inflammation and tissue damage. Neutrophils (brown, bottom). Neutrophils (CD66b^+^) release neutrophil extracellular traps (NETs) and IL-17, which can promote B-cell activation and plasmablast generation. In ANCA-associated vasculitis (AAV), plasmablasts produce anti-neutrophil cytoplasmic antibodies (ANCA) directed against proteinase 3 (PR3) or myeloperoxidase (MPO), which activate neutrophils and drive vasculitic inflammation—exemplifying a direct pathogenic plasmablast–neutrophil axis. Regulatory T Cells (Treg; cyan, lower left). Tregs (CD25^+^FOXP3^+^) normally suppress excessive immune activation through IL-10 and TGF-β secretion. However, in autoimmune diseases, plasmablasts and their precursors may become resistant to Treg-mediated suppression, contributing to loss of immune tolerance and sustained autoantibody production. Regulatory B Cells (Breg; indigo, upper left). Bregs produce IL-10 and share developmental pathways with plasmablasts. Under homeostatic conditions, Bregs exert immunoregulatory functions; however, in autoimmunity, the balance shifts toward pathogenic plasmablast generation at the expense of regulatory populations. Cytokine Milieu Box (lower right). Summary of key cytokines driving plasmablast differentiation: pro-differentiation signals include IL-21 (the principal Tfh-derived driver), IL-6 (inflammatory amplifier), type I IFN (extrafollicular pathway activator), and BAFF/APRIL (survival factors). Regulatory cytokines include IL-10 and TGF-β from Bregs and Tregs. Therapeutic Implications Box (lower left). The network of interactions identifies multiple therapeutic targets: anti-IL-21 strategies to block Tfh signals, JAK inhibitors to interrupt IL-6 and interferon signaling, BAFF/APRIL blockade to reduce DC-derived survival signals, and type I IFN blockade (e.g., Anifrolumab) to suppress interferon-high plasmablast subsets. The convergence of multiple pathways on plasmablast generation provides rationale for combination therapeutic approaches.

Plasmablasts do not function in isolation but engage in complex, bidirectional crosstalk with multiple immune cell populations that profoundly influence their generation, survival, and effector functions [4,5,46]. Understanding these interactions is essential for interpreting plasmablast dynamics in disease and predicting responses to immunotherapy (Figure 3).

T follicular helper (Tfh) cells provide critical support for plasmablast differentiation through IL-21 secretion, CD40L engagement, and ICOS-ICOSL interactions [35,53]. Aberrant expansion of circulating Tfh-like cells correlates with plasmablast levels across multiple autoimmune diseases, suggesting coordinated dysregulation of the T-B cell axis. In SLE, the Tfh–plasmablast interaction is amplified by the interferon-rich milieu, creating a feed-forward loop of B-cell activation [40,41].

Dendritic cells (DCs) and monocytes contribute to plasmablast generation through multiple mechanisms. Type I interferon-producing plasmacytoid DCs (pDCs) are particularly relevant in SLE and pSjD, where their chronic activation sustains the interferon signature that drives extrafollicular plasmablast differentiation [41,42]. Myeloid DCs present autoantigens and provide co-stimulatory signals, while monocyte-derived cells in inflammatory tissues can support local plasmablast survival through BAFF and APRIL production [54].

Regulatory T cells (Tregs) and regulatory B cells (Bregs) normally restrain plasmablast responses, and their dysfunction contributes to autoimmune B-cell hyperactivity [4,55]. Effective immunotherapy may restore regulatory circuits alongside depleting pathogenic plasmablasts, contributing to durable remissions observed with some treatments.

Neutrophils and other innate immune cells interact with plasmablasts through neutrophil extracellular traps (NETs), which expose autoantigens and activate TLRs on B cells. In AAV, this neutrophil–B cell interaction contributes to both ANCA production and disease pathogenesis [56,57].

These cellular interactions are significantly altered by immunotherapies. B-cell depletion with rituximab disrupts T-B cell collaboration and reduces plasmablast generation, while JAK inhibitors interfere with cytokine signaling networks that support plasmablast differentiation [58,59]. Understanding how different therapies reshape the immune cell landscape helps explain variable responses and informs combination treatment strategies.

## 3. Plasmablasts Across Autoimmune Diseases

The following sections summarize disease-specific plasmablast associations. It is important to note that the evidence base varies considerably across diseases. Studies in SLE generally comprise larger, multicenter cohorts with prospective designs, while data in IgG4-RD and AAV often derive from smaller, single-center cross-sectional studies. Where possible, we indicate the strength of evidence supporting each association and should therefore be interpreted in the context of study limitations.

### 3.1. Systemic Lupus Erythematosus

SLE is the best-characterized setting for plasmablast biology, with extensive literature documenting their pathogenic relevance and biomarker utility [19,50,53]. The central role of B cells in SLE pathogenesis is evidenced by the presence of pathogenic autoantibodies, the efficacy of B-cell depletion therapy, and the correlation between B-cell abnormalities and disease activity [49,60]. Multiple studies, including large multicenter cohorts, have demonstrated robust correlations between plasmablast levels and disease parameters [17,19,61]:

Plasmablast surges precede clinical flares by 2–6 weeks, providing a valuable predictive window for intervention [19,62]. Moreover, plasmablast levels correlate with SLEDAI scores, anti-dsDNA antibody titers, complement consumption (low C3/C4), and active nephritis [53,61,63]. These dynamics are driven by an interferon-dependent extrafollicular B-cell activation cascade, linking the type I interferon signature to sustained plasmablastogenesis and B-cell hyperactivity [42,43,44]. Persistent plasmablast elevation identifies patients with refractory disease and a higher risk of cumulative organ damage [64,65]. Therapeutically, the kinetics of B-cell reconstitution following rituximab—particularly early plasmablast reappearance—predict shorter remission duration, with relapse occurring 3–9 months earlier in patients whose plasmablasts return within 4–6 months after treatment [66,67,68]. In contrast, prolonged plasmablast suppression is associated with sustained disease control and reduced glucocorticoid requirements [69,70]. More recently, the therapeutic landscape has expanded substantially, with anti-CD38 therapy (daratumumab) demonstrating notable efficacy in refractory SLE [26] and CD19-directed CAR-T cell therapy achieving unprecedented drug-free remissions in multiply refractory patients [27,71,72]. In lupus nephritis, plasmablast levels correlate with renal activity and proteinuria, and their persistence during treatment predicts incomplete renal response [60,63]. Single-cell and tissue-based studies have further demonstrated plasmablast infiltration within affected kidneys, supporting a direct contribution to local antibody production and immune-mediated tissue damage [49,60].

### 3.2. Sjögren Disease

Sjögren disease (SjD) displays a distinctive plasmablast signature reflecting its unique pathophysiology characterized by glandular lymphocytic infiltration, B-cell hyperactivity, and increased lymphoma risk [47,73]. The disease is marked by chronic stimulation of the B-cell compartment, elevated serum immunoglobulins, and frequent autoantibody production including anti-SSA/Ro and anti-SSB/La [48,74]. In Sjögren’s disease (SjD), plasmablast biology displays distinct but overlapping features with systemic lupus erythematosus. Several studies have shown that plasmablast expansion correlates primarily with systemic disease activity, as measured by the ESSDAI, rather than with sicca symptoms alone [73,75,76]. Elevated plasmablast levels reflect sustained extrafollicular B-cell activation in a BAFF-rich milieu, with circulating BAFF concentrations correlating closely with plasmablast numbers [77,78]. Importantly, persistent plasmablast elevation has been associated with an increased risk of lymphoma development in selected patient subsets, particularly those exhibiting chronic B-cell activation and germinal center–like structures within salivary glands [79,80,81]. At the transcriptional level, circulating plasmablasts in SjD display a pronounced type I interferon signature, mirroring pathways observed in SLE and reinforcing the concept of shared interferon-driven extrafollicular circuits across systemic autoimmune diseases [47,73]. Notably, plasmablasts frequently remain detectable or rapidly reconstitute following rituximab therapy, likely reflecting the persistence of extrafollicular activation foci within salivary glands that are relatively shielded from circulating anti-CD20 antibodies [82,83,84,85,86]. This phenomenon suggests that peripheral blood plasmablast monitoring may underestimate residual tissue-level disease activity and underscores the need for multicompartment assessment strategies in SjD.

Importantly, plasmablasts may persist or rapidly reappear following rituximab therapy in SjD, reflecting ongoing extrafollicular activation and tissue-resident immune niches rather than incomplete B-cell depletion. This observation highlights a key limitation of plasmablasts as universal therapy-response biomarkers. In this context, persistent plasmablast detection should be interpreted as evidence of compartmentalized immune activity rather than treatment failure per se. These findings underscore the context-dependent nature of plasmablasts as biomarkers and the need for disease-specific interpretation frameworks [83,84,85,86,87].

### 3.3. IgG4-Related Disease

In IgG4-RD, plasmablasts represent perhaps the most diagnostically and prognostically informative biomarker available [55,87,88,89]. Unlike other autoimmune conditions where plasmablasts serve as one of several useful markers, in IgG4-RD they occupy a central position in disease assessment and monitoring.

In IgG4-related disease (IgG4-RD), plasmablasts exhibit a set of highly distinctive features that confer exceptional diagnostic and disease-monitoring value. Circulating plasmablast levels are often markedly elevated even when serum IgG4 concentrations remain within the normal range, providing superior sensitivity for disease detection compared with serum IgG4 alone [55,90,91]. Plasmablast frequencies correlate strongly with disease activity as assessed by the IgG4-RD Responder Index, frequently exceeding the performance of serum IgG4 itself as a biomarker (based on prospective cohort studies, though sample sizes remain modest) [55,90]. Clonal analyses have demonstrated oligoclonal plasmablast expansions with restricted immunoglobulin heavy-chain variable region usage, supporting an antigen-driven pathogenic process [56,92]. Therapeutically, plasmablast counts decline rapidly following glucocorticoid or rituximab treatment, and early normalization is predictive of sustained clinical remission [93,94]. The particular utility of plasmablast monitoring in IgG4-RD reflects the well-recognized limitations of serum IgG4, which is normal in up to 30% of histologically confirmed cases and may be elevated in a range of unrelated inflammatory or neoplastic conditions [88,95]. In this context, plasmablast enumeration provides critical complementary diagnostic information and can help resolve clinically ambiguous presentations [55,91,96].

### 3.4. ANCA-Associated Vasculitis

Plasmablasts contribute significantly to the immunopathology of ANCA-associated vasculitis, particularly granulomatosis with polyangiitis (GPA) and microscopic polyangiitis (MPA) [57,58]. The pathogenic role of ANCA—autoantibodies directed against proteinase 3 (PR3) or myeloperoxidase (MPO)—in neutrophil activation and endothelial damage provides a direct link between B-cell–derived antibodies and disease manifestations [58,59].

In ANCA-associated vasculitis (AAV), plasmablast involvement is particularly prominent in PR3-positive disease, where circulating plasmablast levels correlate closely with ANCA titers and markers of immunological activity (based primarily on single-center studies requiring validation) [59,97]. Plasmablast-derived antibodies have been linked to relapse propensity, and persistently elevated plasmablast counts during clinical remission identify patients at increased risk of subsequent disease relapse [97,98]. Multi-omics profiling has revealed enhanced antigen-presentation programs and NF-κB pathway activation in vasculitis-associated plasmablasts, supporting their role as active immunopathogenic effectors rather than passive antibody producers [59]. From a therapeutic perspective, longitudinal monitoring of plasmablast kinetics following rituximab therapy may enable more individualized retreatment strategies in AAV [99,100,101,102,103]. This approach is particularly relevant in granulomatosis with polyangiitis (GPA), where relapse rates remain substantial despite maintenance therapy. In this context, the MAINRITSAN trials have established rituximab as an effective agent for remission maintenance, and integration of plasmablast monitoring may further refine dosing schedules to optimize long-term disease control while minimizing cumulative immunosuppression [102,103].

### 3.5. Rheumatoid Arthritis

RA shows more modest peripheral blood plasmablast elevations compared to SLE, reflecting a disease process primarily centered within synovial tissue rather than diffuse systemic B-cell activation [104,105]. Nevertheless, plasmablasts play a pivotal role in RA, particularly in seropositive disease and in the context of B-cell–depleting therapy [104,106]. Transient plasmablast peaks coincide with synovial disease exacerbation in seropositive RA, correlating with joint inflammation and structural progression [102,106].

Within inflamed synovium, plasmablasts participate in ectopic lymphoid structures (ELS), where local germinal center–like reactions support autoantibody production and perpetuate inflammation [51,52,107]. The rapid reappearance of plasmablasts following B-cell depletion predicts early clinical relapse, mirroring patterns observed in SLE [69,70].

Moreover, anti-citrullinated protein antibody (ACPA) titers correlate with plasmablast-derived antibody production, underscoring their contribution to pathogenic humoral immunity in RA [108,109].ELS formation, present in approximately 40% of patients with RA, creates specialized microenvironments containing follicular dendritic cells, T follicular helper cells, and actively differentiating B cells, thereby sustaining local plasmablast differentiation and tissue-restricted autoimmunity [107,110].

Table 1 summarizes the comparative characteristics of plasmablasts across these five autoimmune diseases, highlighting differences in peripheral expansion, interferon signature, predominant differentiation pathway, disease activity correlation, flare prediction utility, and response to rituximab kinetics monitoring.

## 4. Plasmablasts as Real-Time Biomarkers

### 4.1. Advantages over Traditional Serology

Plasmablasts reflect current immune activation, whereas autoantibody titers represent a historical record produced substantially by long-lived plasma cells [13,14,15]. This fundamental distinction underlies the unique biomarker value of plasmablasts.

Because plasmablasts have exceptionally short lifespans, estimated at approximately 24–72 h, fluctuations in their peripheral blood frequency precede multiple downstream immunological and clinical events [8,17,18]. In several systemic autoimmune diseases, plasmablast surges anticipate overt clinical flares by approximately 2–6 weeks, providing an actionable early warning of impending disease exacerbation [19,62]. These early cellular changes are followed by serologic shifts, including rising autoantibody titers, reflecting the time required for plasmablast-derived antibodies to accumulate in the circulation [17]. Subsequent immune complex formation drives complement consumption, typically manifested by declining C3 and C4 levels [6]. Finally, inflammatory cascades culminate in elevation of acute-phase reactants such as C-reactive protein and erythrocyte sedimentation rate, although this response may be attenuated in interferon-dominant disease states [42].

However, several important caveats must be considered when interpreting plasmablast elevations. Transient plasmablast expansion occurs physiologically following acute infections, vaccinations, and non-disease-related immune activation [18,111]. A recent viral infection can increase plasmablast counts for 1–2 weeks, while vaccination (particularly with mRNA vaccines) may elevate plasmablasts for up to 4 weeks [111]. These confounders can generate false-positive signals if not recognized. Clinicians should ascertain vaccination history and exclude intercurrent infections before attributing plasmablast rises to autoimmune disease activity. Serial measurements demonstrating sustained elevation, rather than single time-point assessments, provide greater specificity for identifying disease-related plasmablast expansion. Additionally, some medications and chronic infections can alter baseline plasmablast levels, necessitating individualized interpretation based on patient context.

This kinetic advantage positions plasmablasts as one of the earliest indicators of loss of disease control. Traditional monitoring strategies relying on quarterly autoantibody titers may miss evolving flares until tissue damage has already occurred [31,32]. Plasmablast monitoring could enable earlier intervention, potentially reducing cumulative organ damage.

### 4.2. Predicting Flare

Numerous studies have identified plasmablast expansion as one of the strongest predictors of disease flare in systemic lupus erythematosus and IgG4-related disease [19,55,90]. In SLE, increases in circulating plasmablasts precede clinical flares more consistently than conventional biomarkers such as anti–double-stranded DNA antibodies, complement consumption, or overt clinical symptoms [61,62]. The reproducible predictive window of approximately 2–6 weeks observed across multiple cohorts provides a critical opportunity for anticipatory, rather than reactive, therapeutic intervention.

**Figure 4:**
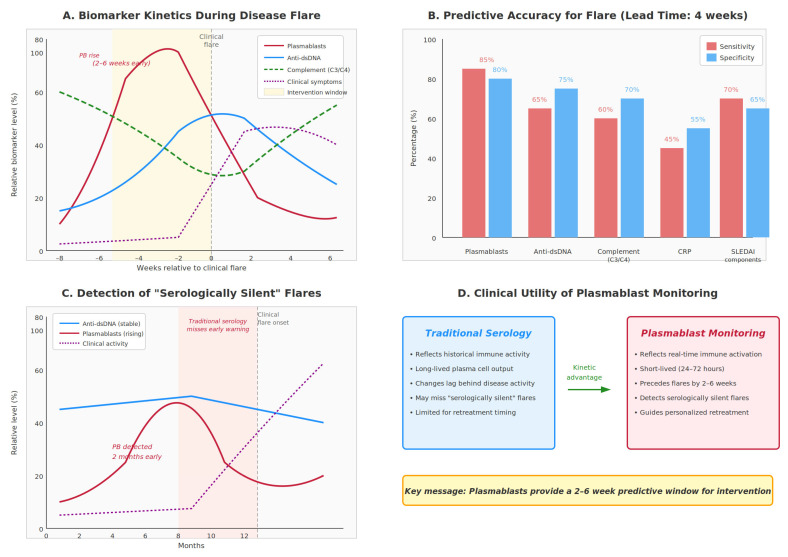
This illustrates the temporal advantage of plasmablast monitoring over traditional serological markers for predicting disease flares in systemic autoimmune diseases. (**A**) Biomarker Kinetics During Disease Flare. Schematic representation of the temporal dynamics of various biomarkers relative to clinical flare onset (time 0, dashed vertical line). Plasmablasts (red solid line) rise 2–6 weeks before clinical manifestations, providing the earliest warning signal. Anti-dsDNA antibodies (blue solid line) increase later, typically peaking at or shortly after flare onset. Complement levels (C3/C4; green dashed line) decrease as immune complexes form, with the nadir coinciding with active disease. Clinical symptoms (purple dotted line) manifest last, after immunological changes have already occurred. The yellow-shaded region indicates the therapeutic intervention window—a 2–6-week period during which preemptive treatment could potentially abort or attenuate the impending flare. This kinetic hierarchy reflects the biological sequence: plasmablast expansion drives new autoantibody production, which subsequently triggers complement consumption and tissue-mediated symptoms. (**B**) Predictive Accuracy for Flare at 4-Week Lead Time. Comparative bar chart displaying sensitivity (red bars) and specificity (blue bars) of various biomarkers for predicting flares with a 4-week anticipation window. Plasmablasts demonstrate superior performance (sensitivity 85%, specificity 80%) compared to anti-dsDNA (65%/75%), complement C3/C4 (60%/70%), C-reactive protein (45%/55%), and composite SLEDAI components (70%/65%). These values represent synthesized estimates from published literature across multiple autoimmune cohorts. The relatively poor performance of CRP reflects its characteristically blunted response in interferon-driven diseases such as SLE. (**C**) Detection of “Serologically Silent” Flares. Time-course illustration of a clinical scenario in which traditional serology fails to predict an impending flare. Anti-dsDNA levels (blue line) remain stable throughout the observation period, while plasmablasts (red line) begin rising at month 4, approximately 2 months before clinical symptoms emerge (month 6, dashed vertical line). Clinical activity (purple dotted line) increases only after plasmablast expansion is well established. The pink-shaded region highlights the period during which traditional serology would provide false reassurance. This scenario, termed “serologically silent” flare, occurs when newly generated plasmablasts produce pathogenic antibodies that drive inflammation before substantially altering total autoantibody titers maintained by long-lived plasma cells. (**D**) Clinical Utility Comparison. Side-by-side summary contrasting traditional serology (left panel, blue) with plasmablast monitoring (right panel, red). Traditional serology reflects historical immune activity derived from long-lived plasma cell output, changes lag behind active disease processes, may miss serologically silent flares, and provides limited guidance for retreatment timing. In contrast, plasmablast monitoring captures real-time immune activation, leverages the short half-life of plasmablasts (24–72 h) to provide dynamic readouts, precedes clinical flares by 2–6 weeks, detects serologically silent flares, and enables personalized retreatment decisions. The green arrow indicates the kinetic advantage conferred by plasmablast monitoring. The bottom annotation emphasizes the key clinical message: plasmablasts provide an actionable 2–6-week predictive window for therapeutic intervention.

Notably, plasmablast monitoring remains informative even during so-called “serologically silent” flares, in which traditional laboratory markers remain stable despite clinical worsening [19]. In these situations, long-lived plasma cells may sustain relatively stable baseline autoantibody titers, whereas newly generated short-lived plasmablasts produce pathogenic antibodies that drive inflammation before significantly altering total antibody levels, thereby explaining the superior sensitivity of plasmablast dynamics in capturing imminent disease activity [17,64] (Figure 4).

### 4.3. Monitoring Response to Therapy

Different immunomodulatory agents influence plasmablast biology through distinct and partially complementary mechanisms, and longitudinal tracking of plasmablast dynamics can therefore provide valuable mechanistic insight into therapeutic response [66,67,68,111]. Rituximab, a CD20-directed monoclonal antibody, effectively depletes mature B cells while initially sparing CD20 low/negative plasmablasts; notably, delayed plasmablast reconstitution beyond 6–9 months following treatment is associated with prolonged clinical remission [66,67,68]. Belimumab, by neutralizing soluble BAFF, preferentially attenuates extrafollicular plasmablastogenesis while relatively preserving germinal center–dependent immune responses [112,113,114]. Janus kinase inhibitors dampen cytokine-driven differentiation pathways, particularly IL-21- and T follicular helper cell-dependent signals that are critical for plasmablast generation [115]. Type I interferon pathway blockade, exemplified by anifrolumab, suppresses interferon-high plasmablast subsets that are especially prominent in systemic lupus erythematosus [45,46]. In contrast to these upstream modulatory approaches, anti-CD38 therapy with daratumumab directly depletes plasmablasts and plasma cells via antibody-dependent cellular cytotoxicity, resulting in rapid suppression of antibody-secreting compartments [26]. These differences underscore that plasmablast kinetics must be interpreted in the context of the therapeutic mechanism of action, rather than as a uniform marker of treatment response.

Beyond their role as biomarkers, plasmablasts actively participate in the cytokine networks that sustain and amplify autoimmune inflammation. Activated plasmablasts secrete not only immunoglobulins but also pro-inflammatory cytokines, including interleukin-6 (IL-6), tumor necrosis factor-α (TNF-α), and lymphotoxin, thereby directly contributing to local and systemic inflammatory circuits [4,54]. Conversely, plasmablast differentiation and survival are exquisitely sensitive to the surrounding cytokine milieu. Key signals such as IL-21, IL-6, type I interferons, B-cell activating factor (BAFF), and a proliferation-inducing ligand (APRIL) collectively promote extrafollicular B-cell activation and plasmablast persistence, establishing self-reinforcing inflammatory loops [34,37,39].

This bidirectional cytokine–plasmablast interplay has important implications for both disease flares and therapeutic complications. Massive plasmablast activation, as observed during severe autoimmune exacerbations or following certain immunomodulatory interventions, may contribute to cytokine release syndrome (CRS)–like phenomena characterized by elevated IL-6, interferon-γ, and other inflammatory mediators [116,117]. Although classical CRS is most frequently associated with CAR-T cell therapy, attenuated cytokine release can accompany vigorous plasmablast responses in autoimmune settings. Awareness of this mechanism may help clinicians anticipate and manage inflammatory complications. Conversely, therapies targeting cytokine pathways—such as IL-6 blockade, Janus kinase inhibition, or interferon receptor antagonism—may exert part of their therapeutic benefit through indirect modulation of plasmablast differentiation, survival, and effector function [44,45,59].

### 4.4. Personalizing Retreatment Schedules

**Figure 5:**
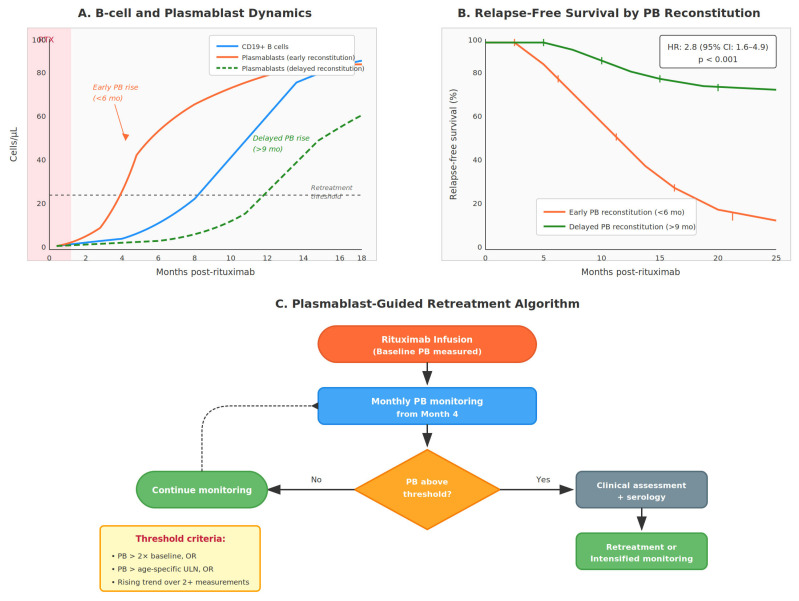
Illustrates B-cell and plasmablast reconstitution dynamics following rituximab and provides a clinical algorithm for biomarker-guided retreatment. (**A**) Reconstitution Dynamics: Early (<6 months) versus delayed (>9 months) plasmablast reconstitution patterns with retreatment threshold. (**B**) Kaplan–Meier Analysis: Relapse-free survival comparing reconstitution patterns (HR 2.8, 95% CI 1.6–4.9, *p* < 0.001). (**C**) Clinical Algorithm: Flowchart from RTX infusion through monthly monitoring to threshold-based retreatment decisions.

The conventional fixed 6-month rituximab retreatment schedule widely used across autoimmune diseases is largely empirical, originating from early clinical trial designs rather than from immunological principles [99,100]. Marked inter-individual variability exists in both the depth and duration of B-cell depletion and in the kinetics of immune reconstitution following therapy [66,116]. Plasmablast-guided retreatment strategies offer a means to address this heterogeneity by avoiding unnecessary early retreatment in patients with sustained B-cell suppression, thereby reducing cumulative immunosuppression, while simultaneously identifying individuals at higher risk of early relapse who may benefit from shortened retreatment intervals [69,70,90,98,102,103]. By incorporating individualized plasmablast thresholds and reconstitution kinetics, this approach shifts B-cell depletion from a rigid, time-based paradigm toward a dynamic precision-medicine strategy tailored to each patient’s immunological trajectory (Figure 5). The feasibility of such tailored dosing has been demonstrated in ANCA-associated vasculitis by the MAINRITSAN2 trial, providing clinical proof-of-concept for biomarker-guided rituximab retreatment [102].

## 5. Therapeutic Targeting of Plasmablasts

### 5.1. CD19-Directed Therapies

CD19 is expressed across the B-cell lineage from early pro-B cells through plasmablasts, but expression diminishes on long-lived plasma cells [29]. This expression pattern makes CD19 an attractive target for depleting the plasmablast compartment while relatively preserving established humoral memory.

Inebilizumab, a humanized monoclonal antibody targeting CD19, induces broad depletion across the B-cell lineage and has been shown to achieve more profound suppression of circulating plasmablast compartments than rituximab, which spares CD19^+^ plasmablasts and plasma cell precursors [30,117,118]. Its clinical efficacy has been most convincingly demonstrated in neuromyelitis optica spectrum disorder (NMOSD), where it is FDA-approved following the N-MOmentum trial, which showed a 77% reduction in attack risk compared with placebo (*p* < 0.0001) [29,119]. Beyond NMOSD, CD19-directed therapy is being actively explored in several antibody-mediated autoimmune diseases, including systemic lupus erythematosus, ANCA-associated vasculitis, IgG4-related disease, and myasthenia gravis [117]. Targeting CD19 may be particularly advantageous in clinical settings where rituximab proves insufficiently effective, such as in patients with persistent plasmablast populations despite apparent depletion of CD20^+^ B cells, highlighting the relevance of plasmablast biology for therapeutic stratification [29,117].

### 5.2. CD38-Directed Depletion

CD38 is highly expressed on plasmablasts and plasma cells, making it an attractive target for depleting antibody-secreting cells. Daratumumab, an anti-CD38 monoclonal antibody initially developed for multiple myeloma, demonstrated notable activity across several refractory autoimmune conditions [26]. Although early reports of anti-CD38 therapy in refractory autoimmune disease are encouraging, available evidence is limited to case series and small observational cohorts. Long-term efficacy, infection risk, and durability of immune reconstitution remain incompletely defined. Consequently, CD38-directed therapy should currently be considered experimental and reserved for highly selected, refractory cases.

In systemic lupus erythematosus, case series and small prospective studies have reported dramatic clinical and serological responses in patients who had failed multiple conventional and biologic therapies (total *n* < 50 patients reported) [26]. However, randomized controlled trial data are currently lacking, and longer-term safety in this population remains to be established

Similar benefit has been described in relapsing or refractory ANCA-associated vasculitis, supporting a role for plasma cell–directed therapy in severe disease (case reports and small series) [120]. In IgG4-related disease characterized by plasma cell–rich tissue infiltrates, daratumumab has been associated with deep and durable responses, further highlighting the pathogenic relevance of antibody-secreting cells in this condition (Limited case reports) [91]. However, depletion of both plasmablasts and long-lived plasma cells carries specific risks, most notably hypogammaglobulinemia and increased susceptibility to infections, necessitating careful monitoring and, in selected cases, immunoglobulin replacement therapy [26,119,121]. The optimal dosing, duration, and patient selection criteria for daratumumab in autoimmune diseases require definition through prospective clinical trials currently in development.

### 5.3. BCMA-Targeted Therapies

B-cell maturation antigen (BCMA, also known as TNFRSF17) is selectively expressed on plasmablasts and plasma cells and plays a critical role in their survival through interactions with BAFF and APRIL [122,123,124]. This restricted expression profile has positioned BCMA as an especially attractive therapeutic target for eliminating autoantibody-producing cells. Several BCMA-directed strategies are currently being explored, including chimeric antigen receptor (CAR) T-cell therapies, bispecific T-cell engagers that redirect cytotoxic T cells via simultaneous binding to BCMA and CD3, and antibody–drug conjugates delivering cytotoxic payloads selectively to antibody-secreting cells [27,72]. Among these approaches, BCMA-directed CAR-T cell therapy has generated particular enthusiasm in systemic lupus erythematosus, where early reports in patients with severe, multiply refractory disease (n < 30 patients in published series) have described profound immunological reset and clinical responses, including apparent drug-free remissions following a single infusion, with durability extending beyond 12 months in some cases [27,71,72]. These preliminary data, while encouraging, derive from single-center case series without randomized comparators and require validation in larger controlled trials.

However, several important considerations temper immediate extrapolation to clinical practice: (1) published data represent highly selected patients from specialized centers; (2) long-term durability beyond 2 years remains unknown; (3) manufacturing requirements, costs, and access barriers limit current applicability; (4) cytokine release syndrome and immune effector cell-associated neurotoxicity syndrome (ICANS), though generally manageable, require intensive monitoring; and (5) infectious complications from prolonged hypogammaglobulinemia require further characterization.

The broader landscape of CAR-T cell target antigens continues to evolve rapidly. Beyond CD19 and BCMA, numerous cancer-specific antigens are under investigation, and insights from hematologic malignancy treatment inform potential applications in autoimmunity [125]. The selection of optimal target antigens—balancing efficacy against autoreactive cells with preservation of protective immunity—remains an active area of research that will shape the future of cellular therapy in autoimmune diseases.

In conclusion, BCMA-targeted approaches, including CAR-T cells, represent a promising but still early therapeutic strategy in autoimmunity. While dramatic responses have been reported in isolated cases, experience remains limited and extrapolation from oncology warrants caution. Antigen specificity, off-target effects, and long-term immune consequences require careful evaluation, as highlighted in recent analyses of cancer-specific target antigens [125].

### 5.4. Combined Strategies and Future Directions

Future therapeutic regimens in systemic autoimmune diseases are likely to rely on rational combinations of agents targeting distinct and complementary aspects of B-cell biology in order to achieve synergistic and more durable disease control [112,113,114,115,125,126]. One promising strategy involves combining B-cell depletion approaches, such as rituximab or anti-CD19 therapies, with blockade of the type I interferon pathway to simultaneously suppress autoreactive B-cell populations and the upstream inflammatory signals that sustain their activation [45,46,127]. Another approach pairs anti-CD38–mediated depletion of plasmablasts and plasma cells with BAFF inhibition, aiming both to eliminate existing antibody-secreting cells and to prevent their regeneration from precursor pools [112,113,114]. Finally, BCMA-directed therapies may be integrated with antigen-specific tolerization strategies to achieve deep, targeted elimination of pathogenic plasma cells while promoting long-term immune reprogramming and sustained immunological tolerance [27,67,68]. Several clinical trials are actively evaluating plasmablast- and plasma cell-targeted therapies in autoimmune diseases. Notable examples include: (1) randomized trials of daratumumab in SLE and systemic sclerosis; (2) phase 2 studies of BCMA-targeted CAR-T cells in lupus nephritis; (3) trials combining rituximab with belimumab (BLISS-BELIEVE, NCT03312907); and (4) studies of next-generation anti-CD19 agents in various autoimmune conditions. These trials will provide crucial data on efficacy, safety, optimal patient selection, and the role of plasmablast monitoring in guiding therapy.

**Figure 6:**
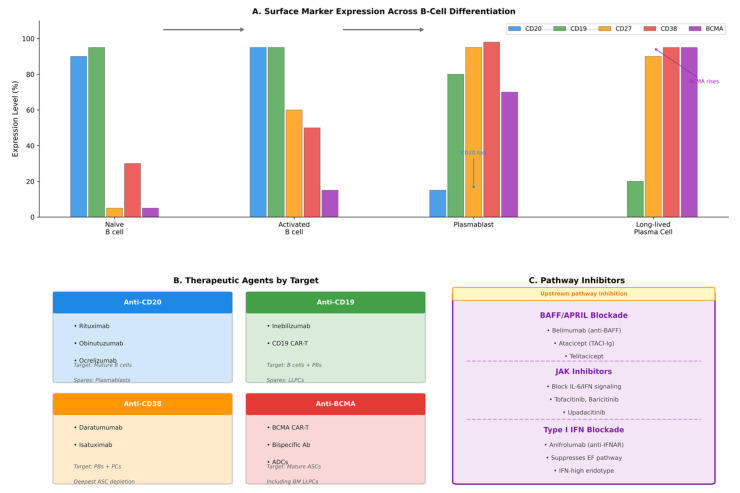
Therapeutic targets on plasmablasts and their precursors. This figure summarizes the dynamic expression of key surface markers across B-cell differentiation stages and the corresponding therapeutic strategies exploiting these patterns to target pathogenic antibody-secreting cells. (**A**) Surface marker expression across B-cell differentiation. Schematic representation of the relative expression (0–100%) of clinically actionable surface markers—CD20, CD19, CD27, CD38, and BCMA—across four differentiation stages: naïve B cells, activated B cells, plasmablasts, and long-lived plasma cells (LLPCs). CD20 is highly expressed on naïve and activated B cells but is markedly downregulated on plasmablasts and absent on LLPCs, explaining the limited efficacy of anti-CD20 therapies against antibody-secreting compartments. CD19 expression persists through the plasmablast stage but decreases on LLPCs, creating a therapeutic window for CD19-directed agents. CD27 and CD38 expression increases with terminal differentiation, peaking on plasmablasts and plasma cells. BCMA expression rises sharply on plasmablasts and reaches maximal levels on LLPCs, making it a key target for eliminating mature and bone marrow–resident antibody-secreting cells. Arrows indicate the direction of B-cell differentiation. (**B**) Therapeutic agents by molecular target. Anti-CD20 agents (blue), including rituximab, obinutuzumab, and ocrelizumab, effectively deplete mature B cells but spare plasmablasts and plasma cells due to CD20 downregulation during terminal differentiation. Anti-CD19 strategies (green), such as inebilizumab and CD19-directed CAR-T cells, achieve broader B-cell depletion encompassing plasmablasts while relatively sparing LLPCs with low CD19 expression. Anti-CD38 monoclonal antibodies (orange), including daratumumab and isatuximab, directly target plasmablasts and plasma cells, resulting in deep depletion of antibody-secreting compartments. Anti-BCMA approaches (red), comprising BCMA-directed CAR-T cells, bispecific T-cell engagers, and antibody–drug conjugates, target mature antibody-secreting cells including bone marrow–resident LLPCs and represent the most comprehensive plasma cell–directed strategies. (**C**) Pathway-level inhibitors of plasmablast generation and survival. BAFF/APRIL pathway blockade with belimumab (anti-BAFF monoclonal antibody), atacicept (TACI-Ig), and telitacicept reduces survival signals for B cells and plasmablasts. Janus kinase (JAK) inhibitors, including tofacitinib, baricitinib, and upadacitinib, interrupt IL-6– and type I interferon–dependent signaling pathways critical for plasmablast differentiation. Type I interferon blockade with anifrolumab (anti-IFNAR) suppresses interferon-driven extrafollicular responses and may be particularly effective in patients with interferon-high plasmablast endotypes. Abbreviations: ADC, antibody–drug conjugate; APRIL, a proliferation-inducing ligand; ASC, antibody-secreting cell; BAFF, B-cell activating factor; BCMA, B-cell maturation antigen; BM, bone marrow; CAR-T, chimeric antigen receptor T cell; EF, extrafollicular; IFN, interferon; IFNAR, interferon-α/β receptor; IL, interleukin; JAK, Janus kinase; LLPC, long-lived plasma cell; PB, plasmablast; PC, plasma cell; TACI, transmembrane activator and calcium-modulator and cyclophilin ligand interactor.

These approaches aim to recalibrate the entire B-cell axis, achieving not merely suppression of autoimmunity but potentially durable tolerance induction [68,115] (Figure 6).

## 6. Integrating Plasmablasts into Clinical Algorithms

### 6.1. Standardization Challenges

Before plasmablast monitoring can be incorporated into clinical guidelines, several methodological challenges require consensus and standardization across centers [31,32,33,119,121]. A major issue relates to gating strategies, as consistent identification of plasmablasts remains difficult due to progressive CD19 downregulation on mature subsets, necessitating harmonized flow cytometry panels and analytical frameworks [31,33]. Marker selection further influences assay performance, with different combinations of CD27 and CD38, and optional inclusion of CD138, BCMA, or Ki-67, affecting sensitivity, specificity, and biological interpretation [32,119]. In addition, lack of standardized reference intervals and the confounding effects of age, sex, ethnicity, and baseline immune status, are major challenges to multicenter comparisons [31,121]. Several confounding factors, including acute infections, recent vaccinations, and certain immunomodulatory therapies, can induce transient plasmablast expansions unrelated to autoimmune disease activity [18,128]. These limitations currently restrict the widespread clinical implementation of plasmablast monitoring and emphasize the need for multicenter harmonization initiatives. Encouragingly, large multicenter harmonization efforts are underway, and international initiatives such as the EuroFlow Consortium and the Human Immunology Project have developed standardized immunophenotyping panels that could be readily adapted for robust and reproducible plasmablast monitoring in clinical practice [32,33].

### 6.2. Practical Algorithm for Clinicians

A pragmatic clinical workflow for integrating plasmablast monitoring into autoimmune disease management can be envisioned across several key stages. At baseline, either at diagnosis or treatment initiation, plasmablast levels should be assessed alongside conventional serological markers, including autoantibodies, complement fractions, immunoglobulin levels, and, when available, interferon activity scores, in order to establish an individual reference profile [31,42,53]. During clinical evaluation of suspected disease flare, repeat plasmablast measurement may provide supportive evidence of active immunological disease when levels rise above the patient’s baseline [19,55,62]. Following rituximab therapy, longitudinal monitoring should be initiated from approximately month 4 onward, with monthly assessments to detect early plasmablast reconstitution that may precede clinical relapse [66,67,68]. Finally, plasmablast kinetics could be incorporated into retreatment decision-making, whereby exceeding an individualized plasmablast threshold, interpreted in conjunction with clinical and serological context, may help guide timely re-intervention and optimize disease control [69,70,102].

### 6.3. Digital Immune Phenotyping and Artificial Intelligence

The use of Artificial intelligence and machine learning approaches offer a powerful framework for integrating plasmablast kinetics with heterogeneous clinical and biological data streams to enhance flare prediction accuracy in autoimmune diseases [53,54]. By combining longitudinal plasmablast measurements with patient-reported outcomes and symptom quantification captured through mobile health applications, wearable-derived physiological and inflammatory signatures such as heart rate variability and activity patterns, and periodic laboratory data including conventional biomarkers and transcriptomic signatures, these models may detect subtle pre-flare states well before overt clinical or laboratory changes become apparent [42,53,54].

Such integrative, data-driven approaches hold the potential to shift autoimmune disease management toward truly preventive medicine, enabling anticipatory therapeutic adjustments days to weeks before clinical deterioration.

## 7. Future Directions in Plasmablast Research

The following directions reflect emerging research trajectories rather than established clinical practice.

### 7.1. Defining Plasmablast ‘Endotypes’

Just as asthma management has evolved from phenotype-based classification toward endotype-driven frameworks that enable targeted therapy selection, autoimmune diseases are increasingly moving toward molecular and immunological stratification [41,53,54]. In this context, plasmablast subsets defined through multi-omics profiling may delineate biologically distinct disease endotypes with direct therapeutic implications. Interferon-high endotypes, characterized by plasmablasts exhibiting strong type I interferon transcriptional signatures, are particularly prominent in systemic lupus erythematosus and Sjögren’s disease and may preferentially respond to interferon pathway blockade [42,43,44,45,46].

**Figure 7:**
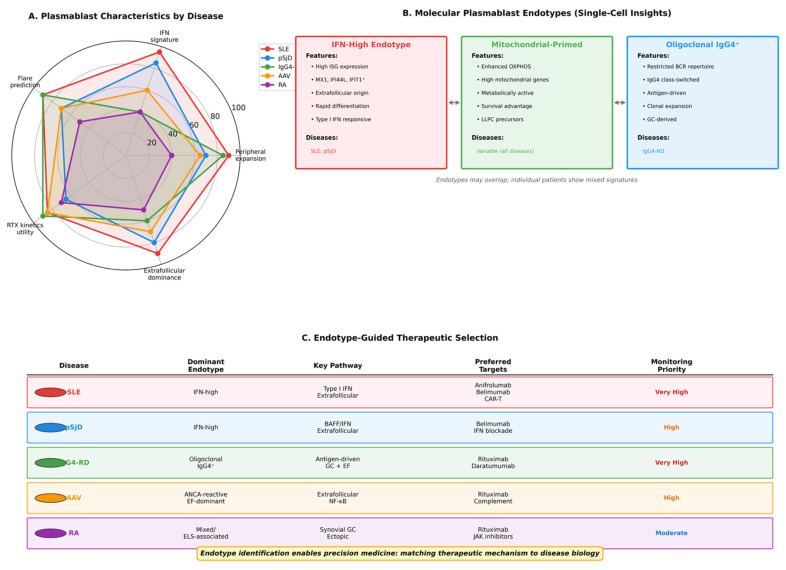
This summarizes disease-specific plasmablast characteristics and introduces the concept of molecular endotypes derived from single-cell technologies, with implications for precision therapeutic selection. (**A**) Plasmablast Characteristics by Disease (Radar Chart). Multi-axis radar plot comparing five key plasmablast features across systemic lupus erythematosus (SLE; red), primary Sjögren disease (pSjD; blue), IgG4-related disease (IgG4-RD; green), ANCA-associated vasculitis (AAV; orange), and rheumatoid arthritis (RA; purple). Axes represent: (1) Peripheral expansion—magnitude of circulating plasmablast elevation; (2) IFN signature—strength of interferon-stimulated gene expression; (3) Flare prediction—utility of plasmablasts for anticipating disease exacerbations; (4) RTX kinetics utility—prognostic value of plasmablast reconstitution timing after rituximab; (5) Extrafollicular dominance—predominance of the extrafollicular differentiation pathway. SLE and IgG4-RD show the most prominent plasmablast involvement across multiple parameters, while RA displays more modest peripheral plasmablast abnormalities consistent with its predominantly synovial pathology. (**B**) Molecular Plasmablast Endotypes. Three distinct plasmablast subsets identified through single-cell RNA sequencing and multi-omics technologies. IFN-High Endotype (red box): Characterized by robust expression of interferon-stimulated genes (ISGs) including MX1, IFI44L, and IFIT1. These plasmablasts arise primarily via the extrafollicular pathway under chronic type I interferon exposure and are particularly prominent in SLE and pSjD. They may respond preferentially to type I interferon blockade. Mitochondrial-Primed Endotype (green box): Defined by enhanced oxidative phosphorylation (OXPHOS) signatures and elevated mitochondrial gene expression. These metabolically active plasmablasts may represent cells with enhanced survival potential or those transitioning towards long-lived plasma cell fate. This endotype is observed variably across autoimmune diseases and may identify patients at risk for treatment-resistant disease. Oligoclonal IgG4^+^ Endotype (blue box): Characteristic of IgG4-RD, featuring restricted B-cell receptor repertoires indicative of antigen-driven clonal expansion. These plasmablasts have undergone class-switch recombination to IgG4 and typically derive from germinal center reactions, suggesting a distinct pathogenic mechanism from the IFN-driven extrafollicular predominance in SLE. The bidirectional arrows between endotype boxes indicate that individual patients may exhibit overlapping signatures, and endotype assignment represents a predominant rather than exclusive molecular phenotype. (**C**) Endotype-Guided Therapeutic Selection. Tabular summary linking disease diagnosis to dominant endotype, key pathogenic pathway, preferred therapeutic targets, and recommended monitoring intensity: SLE: IFN-high endotype; type I IFN/extrafollicular pathway; targets include anifrolumab, belimumab, and CAR-T therapy; very high monitoring priority given strong flare prediction. pSjD: IFN-high endotype; BAFF/IFN-driven extrafollicular pathway; targets include belimumab and IFN blockade; high monitoring priority, though tissue compartmentalization may limit peripheral blood correlation. IgG4-RD: Oligoclonal IgG4^+^ endotype; antigen-driven germinal center and extrafollicular pathway; targets include rituximab and daratumumab; very high monitoring priority given exceptional biomarker utility. AAV: ANCA-reactive extrafollicular-dominant endotype; extrafollicular/NF-κB pathway; targets include rituximab and complement inhibition; high monitoring priority for relapse prediction. RA: Mixed/ectopic lymphoid structure (ELS)-associated endotype; synovial germinal center pathway; targets include rituximab and JAK inhibitors; moderate monitoring priority reflecting predominantly tissue-based pathology. The bottom annotation emphasizes the principle of precision medicine: endotype identification enables matching of therapeutic mechanism to individual disease biology, potentially improving treatment outcomes while minimizing unnecessary immunosuppression.

Oligoclonal IgG4 endotypes, defined by clonally restricted IgG4-producing plasmablast populations, underpin the pathogenesis of IgG4-related disease and highlight the antigen-driven nature of this condition [55,56,92]. Finally, ANCA-reactive extrafollicular endotypes, marked by robust plasmablast involvement and heightened extrafollicular activation, characterize subsets of ANCA-associated vasculitis and provide a mechanistic rationale for targeted B-cell and plasma cell-directed therapies in these patients [57,58,59] (Figure 7).

### 7.2. From Biomarker to Therapeutic Target

Plasmablast-depleting strategies are emerging as a pivotal therapeutic approach for patients with severe and refractory autoimmune diseases, reflecting a paradigm shift toward direct targeting of antibody-secreting cell compartments [26,27,71,72]. Ongoing and upcoming clinical trials evaluating BCMA-targeted antibody–drug conjugates, anti-CD38 monoclonal antibodies, and chimeric antigen receptor (CAR) T-cell therapies are expected to substantially reshape treatment landscapes for refractory autoimmunity. However, several critical questions remain to be addressed, including the optimal depth and duration of plasmablast and plasma cell depletion required to achieve durable remission, strategies to mitigate infectious complications associated with profound humoral immune suppression, and approaches to preserve protective immunity while selectively eliminating autoreactive antibody-producing cells [26,27,71,72,119,122,123,124]. Addressing these challenges will be essential to safely translate plasmablast-directed therapies into routine clinical practice and to fully realize their potential within precision immunology frameworks.

### 7.3. Spatial Immunology and Tissue-Based Assessment

Emerging spatially resolved technologies, including imaging mass cytometry, multiplexed ion beam imaging, and spatial transcriptomics, are providing unprecedented insight into the tissue distribution and functional context of plasmablasts within affected organs in autoimmune diseases [49,54,60]. In Sjögren’s disease, these approaches have revealed plasmablast clustering within periductal regions and ectopic lymphoid structures of salivary glands, supporting a role for local antibody production in glandular pathology [129,130]. In lupus nephritis, tubulointerstitial plasmablast infiltration has been linked to adverse renal outcomes, highlighting the pathogenic relevance of tissue-resident antibody-secreting cells beyond circulating biomarkers [49,60,63]. Similarly, in rheumatoid arthritis, plasmablasts localize within germinal center-like structures in inflamed synovium, reinforcing the concept that ectopic lymphoid microenvironments sustain localized humoral immune responses and contribute to chronic tissue inflammation [51,52,110].

### 7.4. Public Health and Health Economic Implications

Implementing plasmablast monitoring at scale has the potential to generate substantial public health benefits by enabling earlier and more precise intervention in autoimmune diseases. Anticipatory identification of impending flares could reduce cumulative glucocorticoid exposure through timely, targeted treatment adjustments, thereby limiting long-term toxicity. Preventing severe disease exacerbations before clinical deterioration may also decrease hospitalization rates and associated healthcare utilization. In parallel, biomarker-guided strategies could optimize medication use by supporting individualized dosing and retreatment schedules rather than fixed, time-based regimens, ultimately improving therapeutic efficiency while reducing unnecessary immunosuppression [102,103].

## 8. Conclusions

Plasmablasts represent a transformative biomarker at the interface of immunopathogenesis, precision immunology, and clinical decision-making [1,2,3,17,18]. Their rapid kinetics, mechanistic relevance, and predictive value position them as a cornerstone of next-generation autoimmune disease management. As short-lived progeny of activated B cells, plasmablasts provide real-time insight into ongoing immune dysregulation that cannot be obtained from traditional serological monitoring [8,9,10,11,12].

Across systemic lupus erythematosus, SjD, IgG4-related disease, ANCA-associated vasculitis, and rheumatoid arthritis, plasmablast dynamics correlate with disease activity, predict flares, and guide therapeutic decisions [19,55,57,73,104]. The kinetics of plasmablast reconstitution after B-cell–depleting therapy emerges consistently as one of the most powerful predictors of relapse, often surpassing traditional biomarkers in prognostic accuracy [66,67,68,116].

Advances in single-cell technologies have revealed remarkable heterogeneity within the plasmablast compartment, with distinct molecular subsets associated with specific diseases and pathogenic mechanisms [41,53,54]. These insights support endotype-based classification approaches that could guide precision therapy selection. The emergence of plasmablast- and plasma cell-directed therapies including anti-CD38 antibodies and BCMA-targeted agents provides new tools for patients with refractory disease [26,27,71,72].

Several key findings from this review have direct therapeutic implications. First, plasmablast kinetics following B-cell depletion can guide individualized retreatment timing, potentially reducing both under-treatment (premature relapse) and over-treatment (unnecessary immunosuppression). Second, the identification of interferon-high plasmablast subsets supports rational selection of interferon-blocking agents for appropriate patient populations. Third, the persistence of plasmablasts in SjD despite rituximab highlights the need for therapies with enhanced tissue penetration or alternative targets in this condition. Fourth, the remarkable responses to BCMA-targeted CAR-T cells in refractory SLE suggest that deep depletion of antibody-secreting cells may achieve effects beyond those attainable with conventional B-cell depletion. Finally, recognition that plasmablasts participate in cytokine networks—both responding to and producing inflammatory mediators—provides rationale for combination strategies targeting multiple nodes of immune dysregulation.

Implementation challenges remain, including standardization of measurement approaches, establishment of reference intervals, and integration into clinical workflows [31,32,33,119,121]. Nevertheless, the accumulating evidence supports plasmablasts as biomarkers ready for translation from research into clinical practice.

As multi-omic technologies mature and therapeutic targeting improves, plasmablast-guided medicine may evolve into a standard component of personalized care—moving from reactive treatment of established flares to proactive immune monitoring that enables preventive intervention. The shift from static serology to kinetic precision immunology represents a paradigm change with the potential to reduce organ damage, optimize therapy, and improve outcomes for patients with autoimmune diseases.

In summary, plasmablasts represent dynamic, context-dependent biomarkers that capture real-time immune activation across systemic autoimmune diseases. Their greatest clinical value lies not in isolated measurements but in longitudinal monitoring integrated with disease-specific and therapy-specific frameworks. While emerging plasmablast-targeted therapies hold promise, their translation into routine care will require careful patient selection, standardized monitoring, and long-term safety evaluation.

## Figures and Tables

**Table 1:** Comparative characteristics of plasmablasts across autoimmune diseases. Data compiled from referenced studies; strength of evidence varies by disease (see main text for details). References in brackets indicate primary data sources for each value.

Feature	SLE	pSjD	IgG4-RD	AAV	RA
Peripheral plasmablast expansion	+++ [19]	++ [47]	+++ [49]	++ [56]	+ [104]
Type I IFN signature	+++ [41]	+++ [47]	+ [88]	+/++ [56]	+ [104]
Predominant activation pathway	EF [34]	EF [77]	GC/EF [50]	EF > GC [58]	GC/ELS [51]
Correlation with disease activity	SLEDAI [65]	ESSDAI [76]	IgG4-RD RI [49]	BVAS [56]	DAS28 [104]
Flare prediction value	+++ [19]	++ [76]	+++ [91]	++ [99]	+ [70]
Utility for RTX kinetics	+++ [22]	++ [84]	+++ [94]	+++ [102]	++ [70]

Abbreviations: SLE, systemic lupus erythematosus; pSjD, primary Sjögren disease; IgG4-RD, IgG4-related disease; AAV, ANCA-associated vasculitis; RA, rheumatoid arthritis; IFN, interferon; EF, extrafollicular; GC, germinal center; ELS, ectopic lymphoid structures; RI, Responder Index; RTX, rituximab. Symbols: +, mild/modest; ++, moderate; +++, strong/marked.

## Data Availability

No new data were created or analyzed in this study.

## List of Abbreviations

Abbreviation	Full Term
AAV	ANCA-associated vasculitis
ACPA	Anti-citrullinated protein antibody
ADC	Antibody-drug conjugate
APRIL	A proliferation-inducing ligand
ATAC-seq	Assay for Transposase-Accessible Chromatin using sequencing
BAFF	B-cell activating factor
BCMA	B-cell maturation antigen (TNFRSF17)
Blimp-1	B lymphocyte-induced maturation protein-1
Bregs	Regulatory B cells
BVAS	Birmingham Vasculitis Activity Score
C3/C4	Complement components 3 and 4
CAR-T	Chimeric antigen receptor T cell
CD	Cluster of differentiation
CD40L	CD40 ligand
CRP	C-reactive protein
CRS	Cytokine release syndrome
CXCR3/CXCR4	C-X-C chemokine receptors 3 and 4
CyTOF	Cytometry by time of flight (mass cytometry)
DAS28	Disease Activity Score-28 joints
dsDNA	Double-stranded DNA
EF	Extrafollicular
ELS	Ectopic lymphoid structures
ESR	Erythrocyte sedimentation rate
ESSDAI	EULAR Sjögren’s Syndrome Disease Activity Index
EULAR	European Alliance of Associations for Rheumatology
FDA	Food and Drug Administration
GC	Germinal center
GPA	Granulomatosis with polyangiitis
HLA-DR	Human leukocyte antigen-DR
ICANS	Immune effector cell-associated neurotoxicity syndrome
IFI44L	Interferon-induced protein 44-like
IFIT1	Interferon-induced protein with tetratricopeptide repeats 1
IFN	Interferon
Ig	Immunoglobulin
IgG4-RD	IgG4-related disease
IL	Interleukin
IMC	Imaging mass cytometry
IRF4	Interferon regulatory factor 4
ISG	Interferon-stimulated gene
JAK	Janus kinase
Ki-67	Marker of proliferation Ki-67
MIBI	Multiplexed ion beam imaging
MPA	Microscopic polyangiitis
MPO	Myeloperoxidase
MX1	MX dynamin-like GTPase 1
NF-κB	Nuclear factor kappa-light-chain-enhancer of activated B cells
NMOSD	Neuromyelitis optica spectrum disorder
OXPHOS	Oxidative phosphorylation
PR3	Proteinase 3
PRDM1	PR domain zinc finger protein 1
pSjD	Primary Sjögren disease
RA	Rheumatoid arthritis
RI	Responder Index
RNA-seq	RNA sequencing
RTX	Rituximab
scRNA-seq	Single-cell RNA sequencing
SLE	Systemic lupus erythematosus
SLEDAI	SLE Disease Activity Index
SSA/Ro	Sjögren’s syndrome-related antigen A/Ro
SSB/La	Sjögren’s syndrome-related antigen B/La
STAT	Signal transducer and activator of transcription
Tfh	T follicular helper (cells)
TLR	Toll-like receptor
TNF-α	Tumor necrosis factor alpha
TNFRSF17	Tumor necrosis factor receptor superfamily member 17
Tregs	Regulatory T cells
XBP1	X-box binding protein 1
